# Effects of *Bacillus licheniformis* Supplementation on Laying Performance, Antioxidant Capacity, and Cecal Microbiota in Post-Peak Laying Hens

**DOI:** 10.3390/ani16203230

**Published:** 2026-10-14

**Authors:** Kaitian Zheng, Yinglei Xu, Xianci Yao, Yonghao Shi, Chenkai Pan, Yifan Zhong, Haocheng Xu, Caimei Yang

**Affiliations:** College of Animal Science and Technology, College of Veterinary Medicine, Zhejiang Agriculture and Forestry University, Hangzhou 311300, China; 19817120570@163.com (K.Z.); xuyl@zafu.edu.cn (Y.X.); 15639190344@163.com (X.Y.); m19157736914@163.com (Y.S.); h3103149957@163.com (C.P.); yifanzhong@zafu.edu.cn (Y.Z.); haochengxu03@foxmail.com (H.X.)

**Keywords:** *Bacillus licheniformis*, laying hens, laying performance, antioxidant capacity, cecal microbiota

## Abstract

*Bacillus licheniformis*, a multifunctional probiotic, improves performance in peak laying hens, but its effects during post-peak laying—characterized by declining ovarian function, impaired intestinal barrier, and oxidative stress—remain unclear. This study evaluated the effects of dietary *B. licheniformis* on laying performance, antioxidant and immune parameters, intestinal morphology, cecal microbiota, and fermentation metabolites in post-peak-phase laying hens. A total of 720 forty-three-week-old laying hens were randomly assigned to four treatments: a basal diet or the basal diet supplemented with *B. licheniformis* at 2.5 × 10^9^, 5 × 10^9^, or 1 × 10^10^ CFU/kg for 31 days. Supplementation at 5 × 10^9^ CFU/kg consistently improved the laying rate from 78.96 ± 0.47% to 80.40 ± 0.64% (+1.44 percentage points, *p* = 0.0091) and decreased the feed-to-egg ratio from 2.53 ± 0.02 to 2.47 ± 0.02 (−2.4 percentage points, *p* = 0.005). It enhanced systemic antioxidant capacity, as indicated by a decrease in serum MDA (35.8% *p* = 0.002) and increases in GSH-Px (33.2%, *p* = 0.002) and SOD (56.0%, *p* = 0.011). Jejunal and ileal villus height and villus-to-crypt ratio were also increased (*p* < 0.05). Cecal microbiota shifted toward a higher relative abundance of *Clostridia*_UCG-014 (*p* = 0.006) and norank_o_RF39 (*p* = 0.017) and a lower abundance of *Megasphaera* (*p* = 0.009), whereas individual and total VFA concentrations did not differ among groups (*p* > 0.05). In conclusion, 5 × 10^9^ CFU/kg *B. licheniformis* exerted the most consistent benefits in post-peak-phase laying hens, likely through the modulation of gut microbiota, intestinal barrier integrity, and antioxidant status. These findings support *B. licheniformis* as a promising probiotic for sustainable laying-hen production.

## Simple Summary

Post-peak-phase laying hens often experience declining egg production, increased oxidative stress, and poorer gut health. Probiotics may offer a natural way to support hens during this period. In this study, we tested a specific probiotic strain, *Bacillus licheniformis* HJ0135, by adding it to the feed of 43-week-old laying hens at three different doses for 31 days. We found that the middle dose (5 × 10^9^ CFU/kg) provided the most consistent benefits: it improved laying rate and feed efficiency, enhanced antioxidant defenses in blood and liver, and promoted a healthier intestinal structure. The probiotic also shifted the gut microbiota toward more beneficial bacteria such as RF39 and *Shuttleworthia*, and altered cecal metabolites in ways that may improve nutrient use and reduce gut stress. These results suggest that *B. licheniformis* HJ0135 could be a practical, non-antibiotic feed supplement to help maintain health and productivity in post-peak-phase laying hens, supporting more sustainable egg production.

## 1. Introduction

The poultry industry accounts for approximately 40% of global meat production and yields an estimated 100 million tons of eggs annually [1,2]. Demand for eggs continues to grow by approximately 2.57% per year [3]. Better breeding, slaughtering, feeding, and processing technologies have driven the shift toward large-scale, intensive poultry production, encouraging producers to expand farm size and adopt vertically integrated systems [4]. However, antibiotic use has risen in parallel [5], and since these compounds are not fully absorbed or degraded, residues can leak into soil, rivers, and the wider environment [6]. Beyond these direct ecological risks, the continuous selective pressure imposed by antibiotic residues promotes the emergence and enrichment of antibiotic resistance genes and resistant bacteria in the environment [5,7]; this is further aggravated by high-density manure accumulation under intensive systems [8]. Growing environmental and food-safety concerns, together with tightening regulations, have therefore prompted the search for antibiotic alternatives suitable for large-scale production, with probiotic supplementation such as *B. licheniformis* receiving particular attention.

Probiotics have been widely recognized for their beneficial effects on laying-hen production, including improvements in productive performance and egg quality. Dietary supplementation with single or compound probiotic preparations has been reported to increase laying rate and egg weight on average, improve feed conversion efficiency, optimize egg shape index, enhance eggshell quality and albumen height [9], as well as modulate gut microbiota and improve ovarian function [10].

*Bacillus* species are Gram-positive, rod-shaped bacteria with the ability to form endospores. This characteristic endows them with strong resistance to unfavorable environmental conditions. Under adverse conditions, *Bacillus* spp. display remarkable resistance to heat, acidic conditions, and digestive enzymes [11]. Certain *Bacillus* strains also show strong metabolic activity and produce many extracellular enzymes (e.g., amylases, proteases, and cellulases), organic acids, and antimicrobial peptides [12,13]. These strain-specific properties can contribute to nutrient digestion, pathogen inhibition, and host immune enhancement. Beyond the secretion of digestive enzymes and antimicrobial compounds, selected *Bacillus* strains have been documented to regulate intestinal mucosal immunity [14,15] and enhance gut morphology [16]. Consequently, this genus is now used in industrial enzyme production, fermentation, and food additives, and increasingly as a functional, safe feed additive in livestock and poultry [17]. It should be noted that these properties are strain-specific and cannot be generalized.

*B. licheniformis* was originally isolated from soil [18], and has attracted attention for its heat-stable enzymes and diverse antimicrobial substances [19]. The strain used in the present study is a *B. licheniformis* HJ0135 (CGMCC No.9385) suspension (1 × 10^10^ CFU/mL; Zhejiang Vegamax Biotechnology Co., Ltd., Huzhou, China). This strain has been previously shown to alleviate *Clostridium* perfringens-induced intestinal injury in mice by modulating inflammation, inhibiting epithelial apoptosis, and reshaping cecal microbiota and metabolic profiles [20].

Although *B. licheniformis* is widely accepted as an effective probiotic with strong enzyme production and environmental adaptability [18], previous studies have confirmed that dietary *B. licheniformis* can significantly improve production performance and intestinal health in laying hens [21]. The previously characterized strain HJ0135 (CGMCC 9385) was evaluated mainly in a mouse model of C. perfringens infection [20], whereas the present study uses *B. licheniformis* HJ0135 in post-peak-phase laying hens and focuses on production performance, antioxidant status, and intestinal morphology [22]. Similarly, another study showed that *B. licheniformis* DSM5749 improved laying performance and promoted intestinal microecological compensated by increasing the abundance of favorable bacteria and enhancing the expression of tight junction proteins in the intestinal epithelium [23].

Most existing studies on laying hens have focused on the growing period and peak laying phase, whereas physiological studies on hens in the post-peak laying phase remain limited [10,24]. The present study aimed to evaluate the effects of different concentrations of *B. licheniformis* HJ0135 on post-peak laying hens, assessing parameters including egg production performance, hepatic antioxidant capacity, and intestinal microbial communities. These findings provide a basis for developing novel commercial *B. licheniformis* as feed additives.

We hypothesized that *B. licheniformis* HJ0135 would dose-dependently improve laying performance and antioxidant status in post-peak phase hens. Doses of 2.5 × 10^9^ and 5 × 10^9^ CFU/kg were selected based on prior evidence of efficacy in laying hens [23,25], and 1 × 10^10^ CFU/kg was included to test for a plateau effect. Laying rate served as the primary efficacy outcome.

## 2. Materials and Methods

### 2.1. Experimental Animals

The experimental chickens used in this study were laying hens (Fengda No. 1), a strain jointly developed by Anhui Rongda Poultry Industry and Anhui Agricultural University, and officially approved as a new breed by the Ministry of Agriculture in 2009 (Approval No. Agricultural 09 New Breed Certificate No. 72). According to the breed evaluation data, Fengda No. 1 hens reach peak egg production at 23 weeks of age, and the peak period can last for 20 weeks with the laying rate exceeding 80%. Thus, the peak period ends at around 43 weeks of age, after which the laying rate begins to decline. In the present study, the control group showed an average laying rate of 78.96 ± 0.47% over the 4-week experimental period, remaining below the 80% peak threshold, which is consistent with the transition into the post-peak laying phase.

### 2.2. Experimental Design

This study used 720 healthy 43-week-old Fengda No. 1 laying hens as previously described in laying-hen feeding trials [26]. After confirming body weight uniformity, birds were randomly allocated to four groups, each with 20 cages and 9 hens per cage. The groups were: basal diet (B1) and basal diet supplemented with 2.5 × 10^9^ CFU/kg (B2), 5 × 10^9^ CFU/kg (B3), or 1 × 10^10^ CFU/kg (B4) *B. licheniformis*. The ingredient and nutrient levels in the basal diet are shown in Table 1. The experiment lasted 31 days. Eight cages were randomly selected per treatment for subsequent sampling.

### 2.3. Feeding and Management

Birds were fed three times daily at 6:00, 11:00, and 17:00; before each feeding, residual feed from the previous feeding was collected, weighed, and deducted from the feed offered. Each bird received 100 g per feeding, with free access to water. Eggs were manually collected and weighed at 17:00.

Hens were housed in a fully enclosed facility with 15 h of artificial light (6:00–21:00, 20 lux). The ambient temperature was maintained at a range of 20–24 °C using an automatic control system, and manure was removed via a conveyor belt.

*B. licheniformis* preparation was provided by Zhejiang VegaMax Co., Ltd. (Anji, China) Prior to the formal experiment, birds were adapted for 3 days under the same feeding conditions with manual feeding.

### 2.4. Production Performance Measurement and Sample Collection

Daily egg production was recorded, including the number of eggs laid and mean egg weight. Feed conversion ratio, egg qualification rate, and average egg production rate were calculated.

After egg production assessment, eight birds per group were selected randomly, individually numbered, and weighed. Blood was sampled by wing venipuncture and placed directly into tubes without anticoagulant, kept at 4 °C, and centrifuged at 5000 rpm for 10 min to separate serum, which was then stored at −20 °C for biochemical analysis. For histological analysis, we fixed jejunum segments (about 2 cm long) in 4% paraformaldehyde after rinsing them with saline. The birds were euthanized by cervical dislocation first. We also collected liver samples from the upper part, froze them immediately in liquid nitrogen, and stored them at −80 °C for antioxidant assays. Cecal contents were aseptically collected on ice and stored at −80 °C for 16S rRNA sequencing and metabolomic analysis.

### 2.5. Serum Biochemical and Hepatic Antioxidant Analyses

Plasma and liver malondialdehyde (MDA, A003-1-2), total antioxidant capacity (T-AOC, A015-2-1), GSH-Px (A005-1-2), and SOD (A001-3-2) were assayed as oxidative stress parameters (Nanjing Jiancheng Bioengineering Co., Ltd., Nanjing, Jiangsu, China) [27]. Liver samples were homogenized in ice-cold saline (1:9, *w*/*v*) and centrifuged, and the supernatant was used for analysis. Liver oxidative parameters were normalized to total protein concentration, which was determined using a BCA protein assay kit (A045-3-2, Nanjing Jiancheng Bioengineering Co., Ltd., Nanjing, Jiangsu, China). Samples exceeding the linear range were diluted with the provided buffer prior to re-assay. All kits have been widely used for chicken samples.

Plasma levels of immunoglobulins (IgG, H106-1-1; IgA, H108-1-2; IgM, H109-1-1) and cytokines (TNF-α, H052-1-2; IL-1β, H002-1-2; IL-6, H007-1-1; IL-10, H009-1-2) were quantified via ELISA (Nanjing Jiancheng Bioengineering Co., Ltd., Nanjing, Jiangsu, China) according to the manufacturer’s instructions [28]. Target concentrations were calculated against standard curves. Intra- and inter-assay CVs were <10% for all plates.

### 2.6. Jejunum and Ileum Morphology Analysis

From each experimental group, we randomly picked eight birds and euthanized them by cervical dislocation. The entire small intestine was immediately excised under sterile conditions. Jejunum and ileum samples were collected, trimmed into segments, thoroughly rinsed to remove luminal contents, and prepared for histology by dehydration and paraffin embedding as per standard protocols. Hematoxylin and eosin (H&E) staining was performed on the sections, which were then examined under a light microscope.

To quantify intestinal morphology, we used ImageJ (version 1.47, National Institutes of Health, Bethesda, MD, USA). For each tissue section, we randomly selected ten intact villi, then measured villus height (VH) and crypt depth (CD). The villus height-to-crypt depth ratio (VH/CD) was calculated for each villus. The mean of ten measurements per segment was used as the representative value for statistical analysis. All measurements were performed in a blinded manner to minimize observer bias.

### 2.7. 16S rRNA Sequencing and Analysis

DNA Extraction and Amplification. Cecal content samples were collected from eight birds per group (*n* = 8). Total microbial DNA was extracted from cecal contents using a commercial soil DNA kit (Nanjing Jiancheng Bioengineering Institute, Nanjing, China). The V3–V4 hypervariable regions of the 16S rRNA gene were amplified with barcoded primers, and the purified amplicons were used to construct libraries. Sequencing was performed on an Illumina NextSeq 2000 platform (Majorbio Bio-Pharm Technology Co., Ltd., Shanghai, China).

Amplicon sequence variants were labeled with DADA2 in QIIME2 (version 2020.8; online: https://qiime2.org, accessed 15 December 2023), with removal of chloroplast and mitochondrial sequences. Taxonomic assignment was carried out against the SILVA database (v138) via a Naive Bayes classifier.

By applying mothur (v1.30.1), we calculated alpha diversity indices (ACE, Chao1, Shannon, and Simpson) and also generated a Bray–Curtis distance matrix for beta diversity. Group comparisons for alpha diversity were done with the Wilcoxon rank-sum test, while beta diversity patterns were visualized using principal coordinates analysis (PCoA) and statistical significance of group differences was evaluated by PERMANOVA. Differential taxa were identified using LEfSe. LEfSe analysis was conducted using the Kruskal–Wallis test with an LDA score threshold of 2.0. Spearman rank correlation coefficients were first computed. Correlation networks were then visualized using edges corresponding to |r| > 0.6 and statistically there was a difference (*p* < 0.05). All bioinformatics analyses were carried out on the Majorbio Cloud Platform (https://cloud.majorbio.com, accessed on 30 May 2025).

### 2.8. Volatile Fatty Acid (VFA) Analysis

Cecal samples (approximately 0.20 g, *n* = 8 per group) were processed for VFA determination as previously described. Briefly, samples were homogenized in ultrapure water, centrifuged (10 min, 12,000 rpm, 4 °C), and the supernatant was acidified with 25% (*w*/*v*) metaphosphoric acid at 4 °C overnight. After a second centrifugation, the supernatant was filtered (0.22 µm) and subjected to gas chromatography analysis [29].

VFAs, including acetate, propionate, butyrate, the branched-chain acids isobutyrate and isovalerate, plus valerate, were quantified using a GC-FID system (Agilent 7890B, Agilent Technologies Inc., Santa Clara, CA, USA) equipped with a DB-FFAP column (Agilent Technologies Inc., Santa Clara, CA, USA). Quantification was performed using the external standard method.

### 2.9. Metabolomics Analysis

We performed untargeted metabolomics using cecal content samples. Metabolomics profiling was conducted by Majorbio Co., Ltd. (Shanghai, China) using a liquid chromatography–tandem mass spectrometry (LC-MS/MS) platform, following previously reported procedures. Briefly, approximately 50 mg of cecal content was accurately weighed and extracted with 400 μL of pre-cooled methanol–acetonitrile solution (1:1, *v*/*v*). Samples were thoroughly vortexed and subjected to ultrasound extraction at 4 °C for 30 min. To remove insoluble impurities and macromolecules, the mixture was first chilled at −20 °C for 30 min. Centrifugation (13,000 rpm, 4 °C) was performed twice: first for 15 min to collect the supernatant for evaporation under nitrogen, and then for 5 min after reconstitution of the dried residue in 120 μL of acetonitrile–water (1:1, *v*/*v*) and sonication at 5 °C for 5 min. The final supernatant was transferred to vials for LC-MS/MS.

Quality control (QC) samples were analyzed after every eight test samples to monitor instrument stability and analytical reproducibility. Metabolites were detected using an ultra-high-performance liquid chromatography (UPLC) system coupled with a TripleTOF 5600+ high-resolution mass spectrometer (AB SCIEX, Framingham, MA, USA) in both positive and negative electrospray ionization modes.

### 2.10. Statistical Analysis

SPSS (version 25.0, IBM Corp., Armonk, NY, USA) was used for statistical analyses. Individual birds were considered as experimental units. Data are presented as mean ± standard error of the mean (SEM). Means with different lowercase letters differ at *p* < 0.05, and those with different uppercase letters differ at *p* < 0.01.

Group differences were compared using one-way ANOVA combined with Tukey’s post hoc comparisons, and statistical significance was set at *p* ≤ 0.05.

Microbiota data were analyzed as follows: alpha diversity differences were tested with the Wilcoxon rank-sum test; beta diversity was visualized by PCoA ordination on Bray–Curtis dissimilarities, and group differences were tested with PERMANOVA; taxa with differential abundance were identified by LEfSe.

## 3. Results

### 3.1. Egg Production Performance

The effects of various doses of *B. licheniformis* on the laying performance of hens are shown in Table 2. Compared with the basal diet group (B1), the group supplemented with 5 × 10^9^ CFU/kg of the probiotic (B3) showed a significant increase in laying rate (80.40 ± 0.64% vs. 78.96 ± 0.47%, *p* = 0.009). The highest laying rate was observed in the B3 group.

For feed-to-egg ratio, B2 and B3 groups each showed significantly lower levels compared with the control (*p* = 0.006).

### 3.2. Serum Antioxidant, Immune, and Inflammatory Parameters

Effects of dietary B. licheniformis supplementation on serum antioxidant, immune, and inflammatory parameters in laying hens are presented in Table 3. Regarding antioxidant indicators, compared with B1, B3 significantly reduced serum malondialdehyde (MDA) levels (2.15 ± 0.20 vs. 1.38 ± 0.08, *p* = 0.002) and elevated glutathione peroxidase (GSH-Px) activity (641.41 ± 90.15 vs. 854.65 ± 41.37, *p* = 0.002).

In terms of inflammatory cytokines, the B3 group exhibited a significant reduction in serum tumor necrosis factor-alpha (TNF-α) concentration relative to other groups (44.75 ± 5.31 vs. 24.98 ± 2.09, *p* = 0.002).

### 3.3. Hepatic Antioxidant Indicators

Antioxidant capacity was determined in liver samples (Table 4). Compared with B1, B3 exhibited a significant reduction in MDA content, along with remarkable increases in SOD and GSH-Px activities.

### 3.4. Intestinal Morphology Analysis

As shown in Table 5, dietary supplementation of 5 × 10^9^ CFU/kg *B. licheniformis* significantly increased VH and VH/CD in both the jejunum and ileum (*p* < 0.05), indicating that moderate addition of *B. licheniformis* to the daily diet is beneficial to the intestinal structure, which may facilitate nutrient absorption.

### 3.5. Volatile Fatty Acid Levels

Cecal VFAs showed a numerical trend toward higher acetate and propionate and lower branched-chain fatty acids in B3 than in B1, although these differences were not statistically significant (Figure 1, *p* > 0.05). Given the limited sample size, these data should be considered descriptive.

### 3.6. Cecal Microbiota Diversity and Community Composition

#### 3.6.1. Alpha Diversity Analysis

Alpha diversity analysis of the cecal microbiota, including Chao1 and Shannon indices, showed that supplementation with *B. licheniformis* HJ0135 had no significant effects on microbial richness or diversity (Figure 2). These results suggest that *B. licheniformis* supplementation did not alter the overall diversity of the microbial community under the present experimental conditions.

#### 3.6.2. Beta Diversity Analysis

Beta diversity analysis based on Bray–Curtis distances showed clear separation among groups (Figure 3). PERMANOVA further confirmed that microbial community structure was significantly affected by dietary *B. licheniformis* supplementation (R^2^ = 0.1708, *p* < 0.001).

#### 3.6.3. Microbial Community Composition

At the phylum level, Firmicutes (also referred to as Bacillota in the updated taxonomy) and Bacteroidota were identified as the dominant bacterial phyla across all groups (Figure 4). Dietary supplementation with different levels of *B. licheniformis* HJ0135 was associated with shifts in the relative abundance of major phyla. Specifically, B3 exhibited a lower proportion of *Bacteroidota* and a higher proportion of *Firmicutes* compared with the control group, resulting in an altered *Bacteroidota*/*Firmicutes* ratio.

At the genus level, the microbial composition also varied among treatment groups (Figure 5). The dominant genera included *Lachnospiraceae*, *Faecalibacterium*, *Bacteroides*, *Rikenellaceae*_RC9_gut_group, and no_rank_o_RF39 (hereafter referred to as RF39), which are widely recognized as key members involved in carbohydrate fermentation and amino acid metabolism [30]. With *B. licheniformis* supplementation, commensal genera such as RF39 showed an increasing trend, whereas certain genera potentially associated with pathogens showed a decreasing trend.

#### 3.6.4. LEfSe Analysis of Differential Microbial Taxa

LEfSe analysis identified multiple microbial taxa that differed significantly among treatment groups (Figure 6). B3 showed enrichment of several taxa such as RF39 and no_rank_o_*Clostridia*_UCG-014.

### 3.7. Overview of Metabolite Profiling and Data Quality Assessment

A total of 26,457 ion features were detected by LC-MS analysis, including 13,858 in positive mode and 12,599 in negative mode. Among these, 3683 metabolites were successfully identified, of which 2540 were annotated in public databases and 1099 were mapped to KEGG pathways. Multivariate statistical analysis was performed to evaluate global metabolic differences between groups (Table 6). PCA revealed a separation trend between groups B1 and B3; however, PERMANOVA indicated that the overall metabolic profiles did not differ significantly between the two groups (R = 0.2966, *p* = 0.584; Figure 7). In addition, quality control (QC) samples (*n* = 3) clustered tightly in the PCA score plot, indicating good analytical stability and reproducibility.

To further characterize metabolic alterations induced by B3 supplementation, partial least squares discriminant analysis (PLS-DA) was performed between the B1 and B3 groups. The PLS-DA score plot showed a clear separation between the two groups, indicating a distinct metabolic phenotype associated with B3 treatment (Figure 8a). The model explained 23.3% of the X variance (R^2^X(cum) = 0.233) and 96.0% of the group variance (R^2^Y(cum) = 0.96), with a cumulative predictive ability of Q^2^(cum) = 0.506. Permutation testing (200 permutations) confirmed that both R^2^Y and Q^2^ were significantly higher than those of permuted models (R^2^Y: *p* < 0.005; Q^2^: *p* = 0.005), indicating that the model was valid and not overfitted. Between B3 and B1, 466 metabolites were detected as significantly differential (Figure 8b). Among these, 205 were elevated and 261 were reduced in the B3 group.

Metabolic pathway analysis based on KEGG/HMDB annotation revealed that several pathways were significantly enriched. In the metabolite-associated pathways (Ms), tryptophan metabolism, caffeine metabolism, and taurine and hypotaurine metabolism were significantly altered (*p* < 0.05). In the enzyme/interaction-associated pathways (EIPs), ABC transporters and neuroactive ligand–receptor interaction were notably enriched (Figure 9).

In terms of cofactor and vitamin metabolism, folate biosynthesis, biotin metabolism, Vitamin B6 metabolism, and nicotinate and nicotinamide metabolism were positively enriched in the B3 group. In addition, pantothenate and coenzyme A biosynthesis and biosynthesis of cofactor pathways also showed significantly elevated DA scores.

In contrast, starch and sucrose metabolism and taurine and hypotaurine metabolism exhibited negative DA scores in the B3 group, indicating a relative downregulation of specific carbohydrate metabolism and sulfur-containing amino acid-related pathways.

Spearman correlation analysis was conducted to evaluate associations between significantly differential metabolites and dominant bacterial genera (Figure 10). The results showed that multiple metabolites were significantly correlated with specific bacterial taxa (|r| > 0.4, *p* < 0.05). Specifically, *Shuttleworthia* and RF39 showed a positive correlation with several lipid-related metabolites, such as Lyso-PC (18:0) and LPE (18:1). In contrast, g_*Rikenellaceae*_RC9_gut_group exhibited significant negative correlations with amino acid metabolites including L-proline and L-valine (Table 7).

## 4. Discussion

Consistent with previous reports in peak laying hens, dietary *B. licheniformis* increased egg production from 94.0% to 98.4% (a 4.4 percentage-point improvement) [23]. In the present study, dietary *B. licheniformis* improved the laying rate from 78.96% to 80.40% and reduced the feed-to-egg ratio from 2.53 to 2.47 in post-peak-phase laying hens. The smaller absolute gain compared with peak laying studies likely reflects the lower baseline and declining physiological capacity of post-peak-phase laying hens, yet the improvement remains practically relevant given that feed costs dominate total production costs in this stage. The present study further reveals an enrichment of RF39 (LDA = 3.66, *p* = 0.037) in the cecal microbiota, a genus linked to short-chain fatty acid production, and this enrichment was associated with changes in cecal metabolites, suggesting a potential microbial–metabolic interaction [31]. However, because the correlation analysis was limited to microbiota and metabolites, whether these changes contribute to the observed performance benefits remains to be established.

Beyond intestinal morphology, the RF39-enriched microbiota were also coupled with a systemic antioxidant shift. Serum GSH-Px and SOD were both elevated, while serum and hepatic MDA declined correspondingly. Consistent with previous findings from our laboratory, dietary Bacillus supplementation can enhance antioxidant enzyme activities by activating the Nrf2 signaling pathway [32]. Mechanistically, the Nrf2/Keap1 pathway represents the master regulatory axis for cellular antioxidant defense: under oxidative stress, Keap1 cysteine residues are modified, allowing Nrf2 to translocate to the nucleus and bind antioxidant response elements (AREs), thereby upregulating SOD and GSH-Px transcription [33]. In addition, *B. licheniformis* itself produces H_2_S, which can chelate Fe^2+^ and protect against ROS damage [34]. In the present study, we propose a dual mechanism to explain the antioxidant shift observed in the B3 group. On the host side, the coordinated elevation of serum GSH-Px and SOD, together with the decline in serum and hepatic MDA, is consistent with the activation of the Nrf2/Keap1-ARE axis. On the microbial side, the strain’s intrinsic H_2_S-mediated Fe^2+^ chelation may suppress Fenton-type hydroxyl radical generation and reduce intracellular ROS accumulation. These coordinated changes suggest that *B. licheniformis* HJ0135 strengthened antioxidant defenses, potentially countering the oxidative burden that accumulates during the post-peak laying period.

In this study, the addition of *B. licheniformis* to the diet did not significantly alter the overall structure of the cecal microbiota in laying hens, as indicated by PERMANOVA (R^2^ = 0.104, *p* = 0.471), in which 89.6% of the total variation was attributed to within-group differences. However, the relative abundance of specific taxa was significantly affected. At the genus level, the abundance of Campylobacter was significantly higher in the control group (B1) than in the B3 group (*p* = 0.032; FDR-adjusted *p* = 0.039), which is mainly recognized as a conditional pathogenic bacterium associated with intestinal inflammatory responses and diarrhea and can disrupt intestinal microbial homeostasis and impair gut barrier function. The *Rikenellaceae*_RC9_gut_group (LDA score = 5.02) and RF39 (LDA score = 3.66) were enriched in the B3 group, which likely contribute to amino acid and energy metabolism [35], thereby promoting gut health and host metabolic efficiency [36]. LEfSe analysis identified RF39 as a key discriminatory biomarker characterizing the *B. licheniformis*-supplemented group (*p* = 0.037), reflecting its statistically robust enrichment [35]. These parallel trends between RF39 enrichment and the increased serum IgG (*p* = 0.040) and IgM (*p* = 0.012) levels observed in the B3 group suggest that this genus may play a potential immunomodulatory role under probiotic intervention. Although the specific immunomodulatory mechanism of this uncultured taxon remains to be elucidated, its enrichment paralleled changes in several metabolic pathways (e.g., folate biosynthesis, biotin metabolism, Vitamin B6 metabolism, and nicotinate and nicotinamide metabolism) [35], suggesting a potential indirect role that warrants further investigation.

Studies have indicated that microbial fermentation yields VFAs that may provide a range of approximately 10–15% of basal metabolic energy in mammals [37]. Moreover, VFAs also act as signaling molecules between the gut microbiota and host metabolism [38]. Studies on poultry have established a clear correlation between RF39 abundance and elevated VFAs levels [39]. In our previous study, we proposed that VFAs are important microbial metabolites whose levels may be largely regulated by changes in microbiota composition [32]. Consistent with this, other studies have shown that, in laying hens, VFAs improve gut barrier function, regulate microbial communities, and enhance immune function [40]. Consistent with these functions, an upward trend in acetate was observed in the B3 group. Although the VFA differences did not reach statistical significance in this study, VFA production remains a plausible mechanistic link between *B. licheniformis* supplementation and the observed improvements in laying performance. In the present study, several findings are consistent with this proposed mechanism: RF39, an obligate fermenter that produces acetate and H_2_ from residual carbohydrates, was significantly enriched in B3 (LDA = 3.66, *p* = 0.037); the B3 group showed a numerical upward trend in acetate; and B3 exhibited increased serum IgG (*p* = 0.040) and IgM (*p* = 0.012), improved villus height (VH) and villus-to-crypt ratio (VH/CD) (Table 5, *p* < 0.05). Together, these observations support the possibility that B. licheniformis supplementation may enhance microbial fermentation and VFA production, thereby contributing to improved intestinal barrier function, immune status, and nutrient utilization in post-peak laying hens. However, because individual and total VFAs did not differ significantly among groups, this pathway should be regarded as a potential mechanism rather than a confirmed mediator.

At the metabolic level, the gut environment enriched with RF39 and *Shuttleworthia* was accompanied by increased levels of L-proline and the lysophospholipids Lyso-PC (18:0) and Lyso-PE (18:0). L-proline is a key amino acid involved in protein synthesis and intestinal energy supply, while lysophospholipids are bioactive lipid mediators that contribute to membrane remodeling and epithelial repair. RF39, an uncultured lineage of *Tenericutes*, possesses a highly reduced genome and limited metabolic capacity [41]. Its expansion in the gut likely reflects a simplified trophic network in which this low-capacity bacterium acts as an obligate fermenter, producing acetate and H_2_ from residual carbohydrates [42]. Such fermentation end-products can serve as substrates for cross-feeding with other members of the gut community, potentially contributing to the observed shifts in amino acid and lipid metabolism. However, the causal relationships between RF39 enrichment, the altered metabolite profile, and the improved performance of laying hens remain to be established. Future studies using metagenomic and metabolomic integration, together with targeted validation of key metabolites, will be needed to clarify whether these microbial and metabolic changes directly mediate the probiotic effects of *B. licheniformis*.

*Shuttleworthia*, by contrast, is a saccharolytic and proteolytic genus strongly associated with propionate, acetate, and butyrate production in the hindgut [43]. *Shuttleworthia* abundance has been shown to be positively connected with microbial protein synthesis (particularly proline and hydroxyproline). Simultaneously, the increase in lysophospholipids (Lyso-PC and Lyso-PE) likely reflects enhanced phospholipid turnover at the intestinal brush-border membrane, possibly driven by probiotic-mediated changes in bile acid secretion and micellar lipid processing [44,45]. Altogether, the dual enrichment of RF39 and *Shuttleworthia* creates a gut metabolic environment characterized by reduced metal-chelating interference.

Lyso-PC (18:0) serves as a natural ligand for the nuclear receptor PPAR-γ [46]. Its elevation activates fatty acid oxidation and energy expenditure programs in the intestine and adipose tissue, thereby markedly reducing body fat deposition and improving systemic glucose tolerance. This metabolic reprogramming essentially redirects energy that would otherwise be stored as fat toward the reproductive system, facilitating yolk precursor synthesis. In parallel, Lyso-PE (18:0) potently stimulates GLP-1 secretion by activating the GPR119 receptor on intestinal L cells. The rise in GLP-1 not only boosts glucose-stimulated insulin secretion and glycemic utilization, but also improves hepatic glucolipid metabolism [47]. In laying hens, these shifts suggest that absorbed nutrients get directed toward energy-intensive processes—follicle development and egg white protein synthesis—rather than being lost to fat deposition or excess heat production. These findings suggest a coordinated mechanism: *B. licheniformis* enriches beneficial microbiota (especially RF39), the consequent remodeling of the gut metabolic milieu (reduced chelators, elevated proline and lysophospholipids), optimizes intestinal structure, and ultimately improves nutrient use (such as better FCR).

The magnitude of the probiotic effect should also be viewed in the context of dose, as the benefits of *B. licheniformis* do not increase monotonically with supplementation level. Lei et al. evaluated six dietary levels (0, 0.01, 0.02, 0.03, 0.06, and 0.09%) in laying hens and observed clear non-linear dose responses. The highest egg production (98.4%) occurred at 0.01%, whereas 0.02, 0.03, and 0.09% produced intermediate values (96.1–96.6%), and 0.09% showed the least improvement in eggshell thickness and strength among all supplemented groups [22]. Albumen height increased significantly only at 0.03 and 0.06%, Haugh units improved only at 0.06%, and the deepest crypts were recorded at 0.06%, even deeper than the control, despite the same group having the greatest villus height [23]. These findings indicate that different endpoints may have different optimal doses, and that exceeding a certain level can diminish or even reverse the expected benefit.

Several non-exclusive mechanisms may explain the non-monotonic dose response, including intensified competition for adhesion sites and nutrients, excessive accumulation of fermentation acids, saturation of host recognition pathways, and a shift toward proteolytic metabolism at high doses [48]. In a gnotobiotic pig model, low-dose L. acidophilus promoted protective IFN-γ-producing T-cell responses, whereas high-dose administration induced a qualitatively different response characterized by strong Treg cell activation and increased IL-10 and TGF-β production, which suppressed protective immunity [49]. A similar phenomenon has also been reported in laying hens [50]. However, some studies have reported that high probiotic doses do not necessarily impair production performance [51], which may reflect the cumulative outcome of multiple interacting factors. In the present study, three supplementation levels were tested. The intermediate dose (5 × 10^9^ CFU/kg, B3) produced the most consistent improvements in laying rate, feed-to-egg ratio, antioxidant capacity, and intestinal barrier function, while the low and high doses also showed beneficial but less pronounced effects. These results were directionally consistent with the moderate-dose benefits reported by Lei et al. [22]. However, because only three doses within a limited range were tested, the existence of an optimal dose–response window for *B. licheniformis* HJ0135 cannot be established from our data. Future studies should include a broader range of supplementation levels to define the dose–response curve and to determine whether the dose maximizing laying performance is also optimal for intestinal morphology, antioxidant status, and egg quality.

## 5. Conclusions

In summary, dietary supplementation with *B. licheniformis* HJ0135 at 5 × 10^9^ CFU/kg was associated with improved laying performance, feed efficiency, intestinal morphology, and antioxidant capacity in post-peak-phase laying hens, accompanied by a shift in cecal microbiota composition, notably the enrichment of RF39 and *Shuttleworthia*. These microbial changes co-occurred with altered cecal metabolites, including increased L-proline and lysophospholipids, suggesting a potential microbial–metabolic mechanism underlying the observed benefits. However, this study was limited to a 4-week trial and did not establish causal relationships among microbiota, metabolites, and performance. Future studies with longer durations, dose–response designs, and metagenomic–metabolomic integration are needed to validate these findings and support the use of *B. licheniformis* as a non-antibiotic strategy for sustainable egg production.

## Figures and Tables

**Figure 1:**
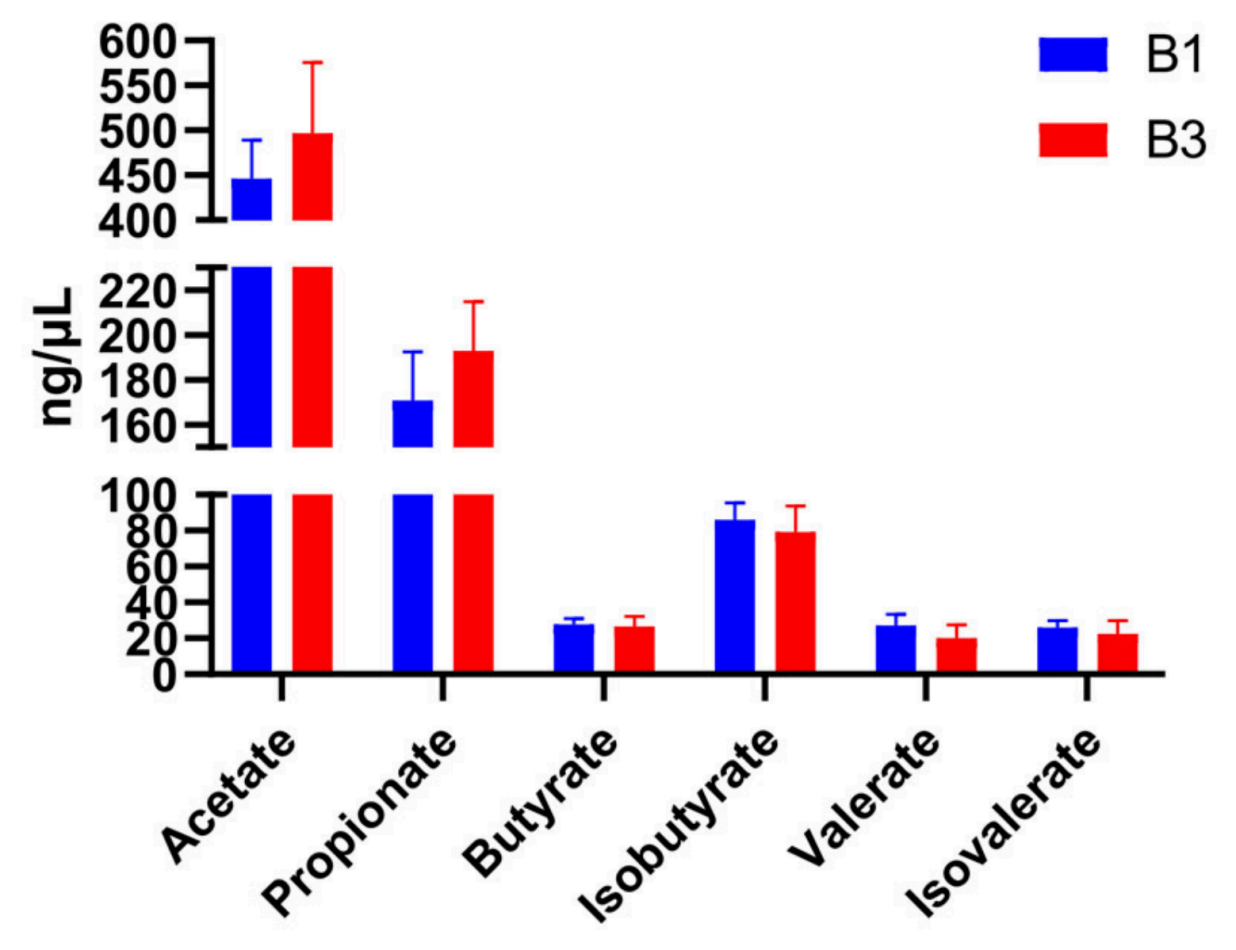
Effects of dietary *B. licheniformis* supplementation on VFA concentrations in laying hens.

**Figure 2:**
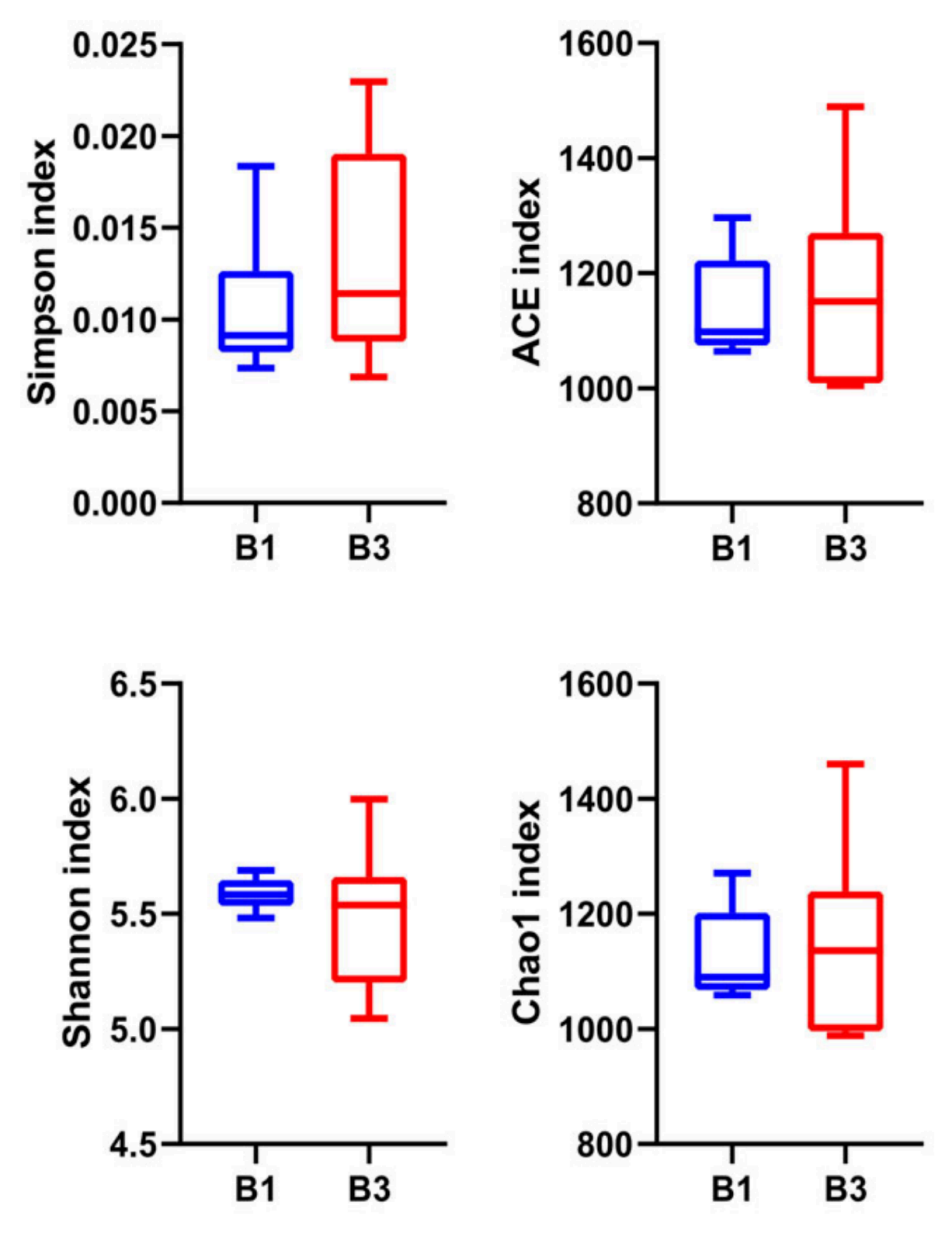
Effects of dietary *B. licheniformis* supplementation on cecal microbial α-diversity in laying hens.

**Figure 3:**
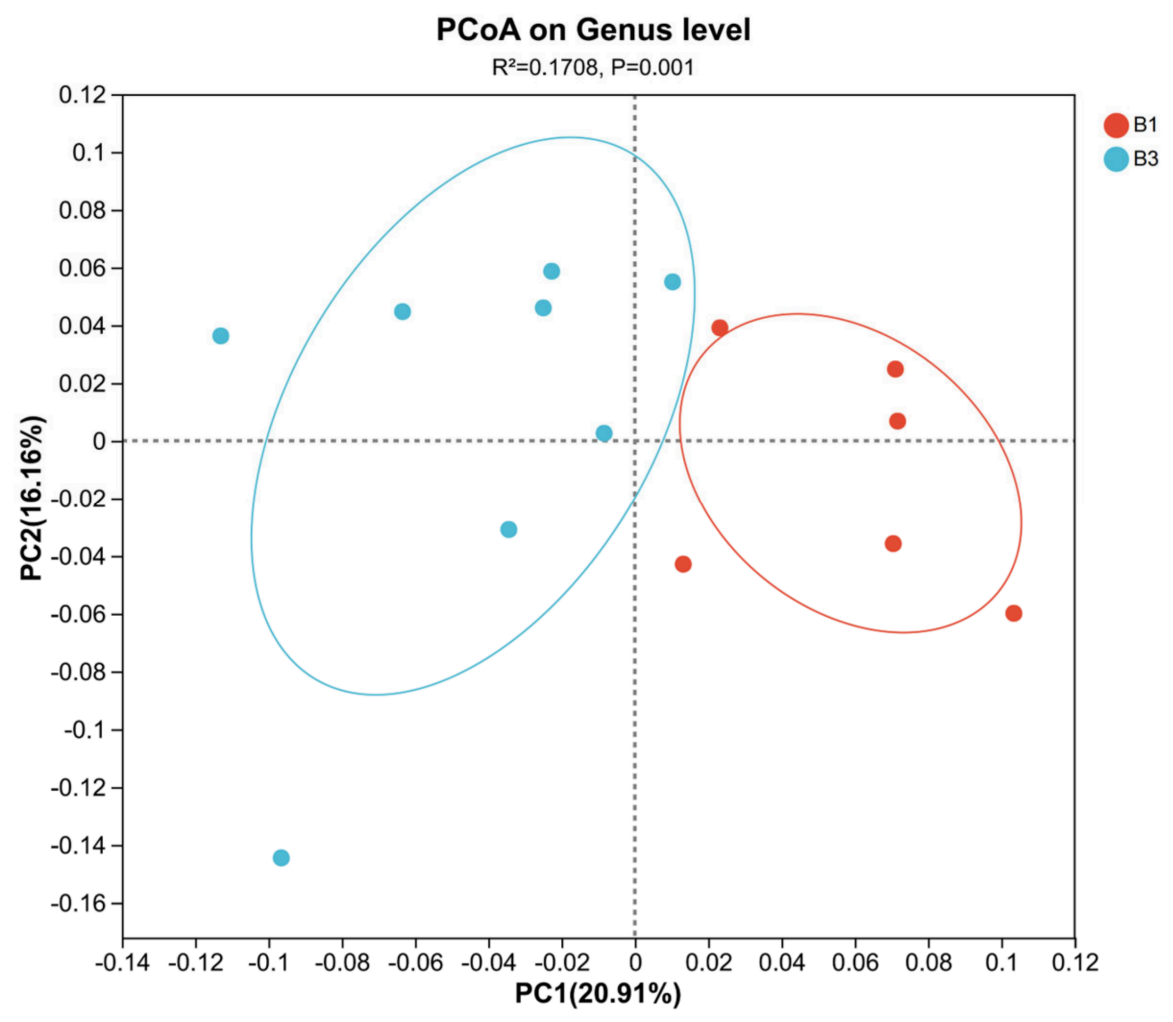
Principal coordinate analysis (PCoA) of cecal microbial communities based on Bray–Curtis distances in laying hens fed different doses of *B. licheniformis*.

**Figure 4:**
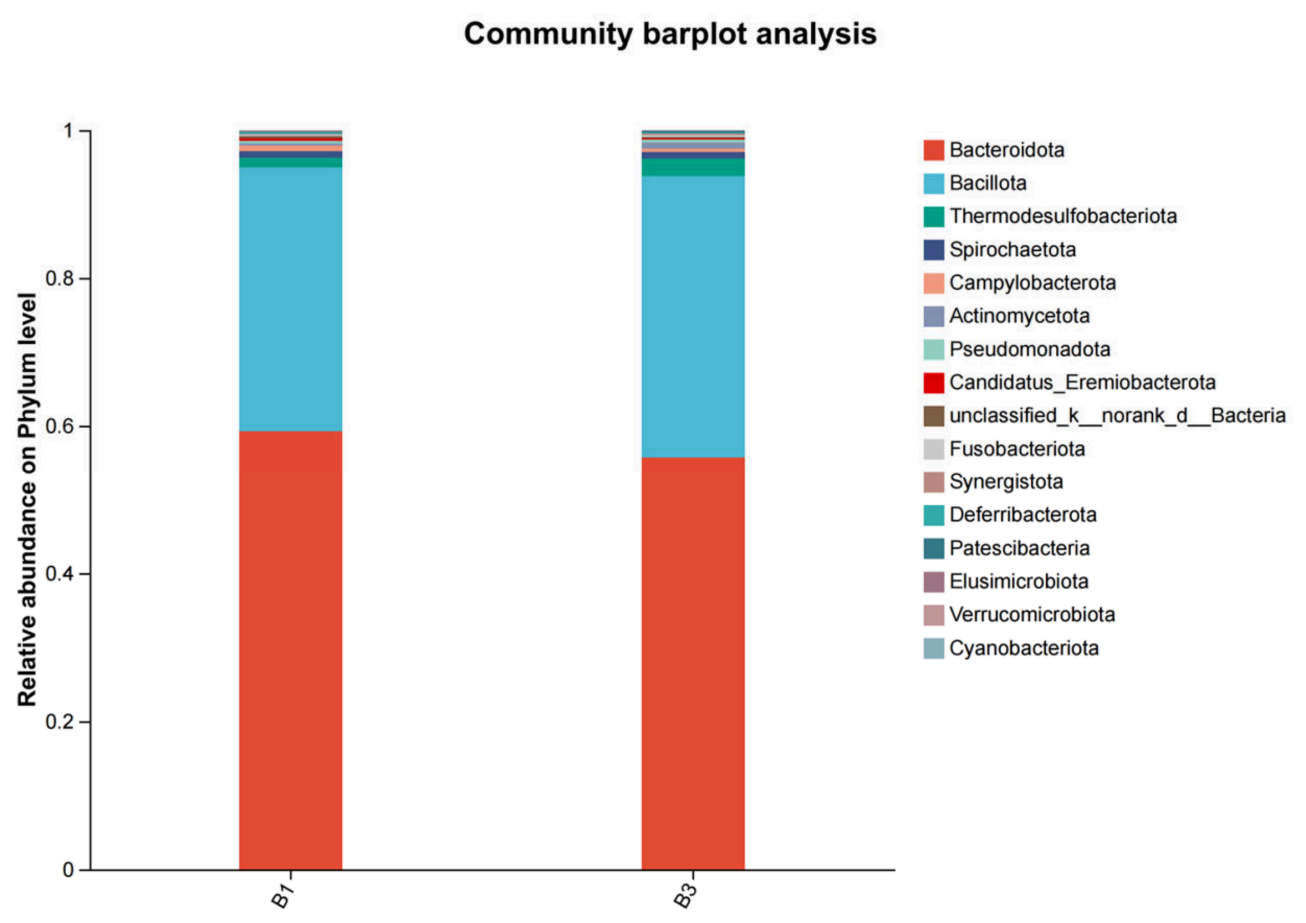
The microbial community at the phylum level.

**Figure 5:**
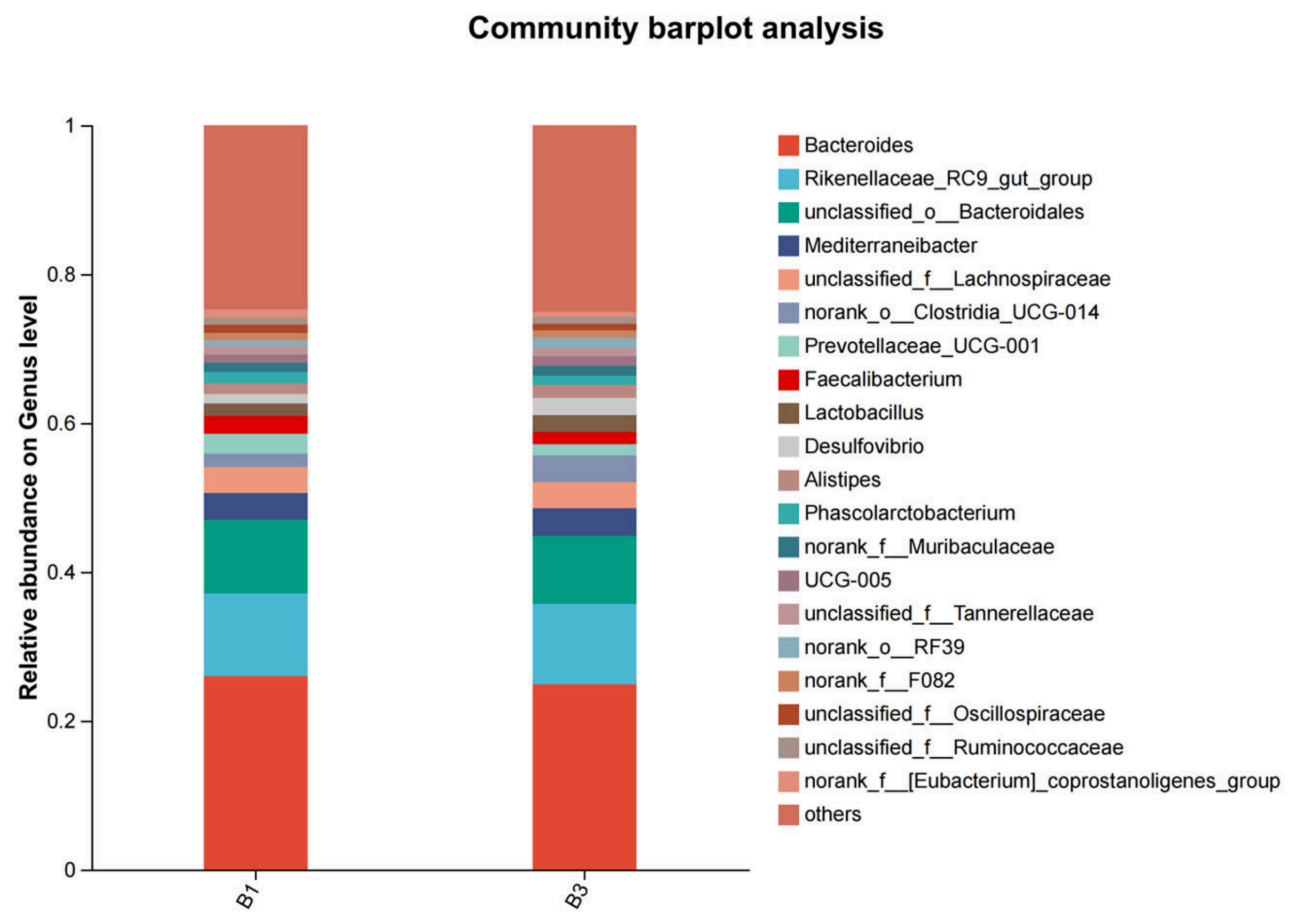
The microbial community at the genus level.

**Figure 6:**
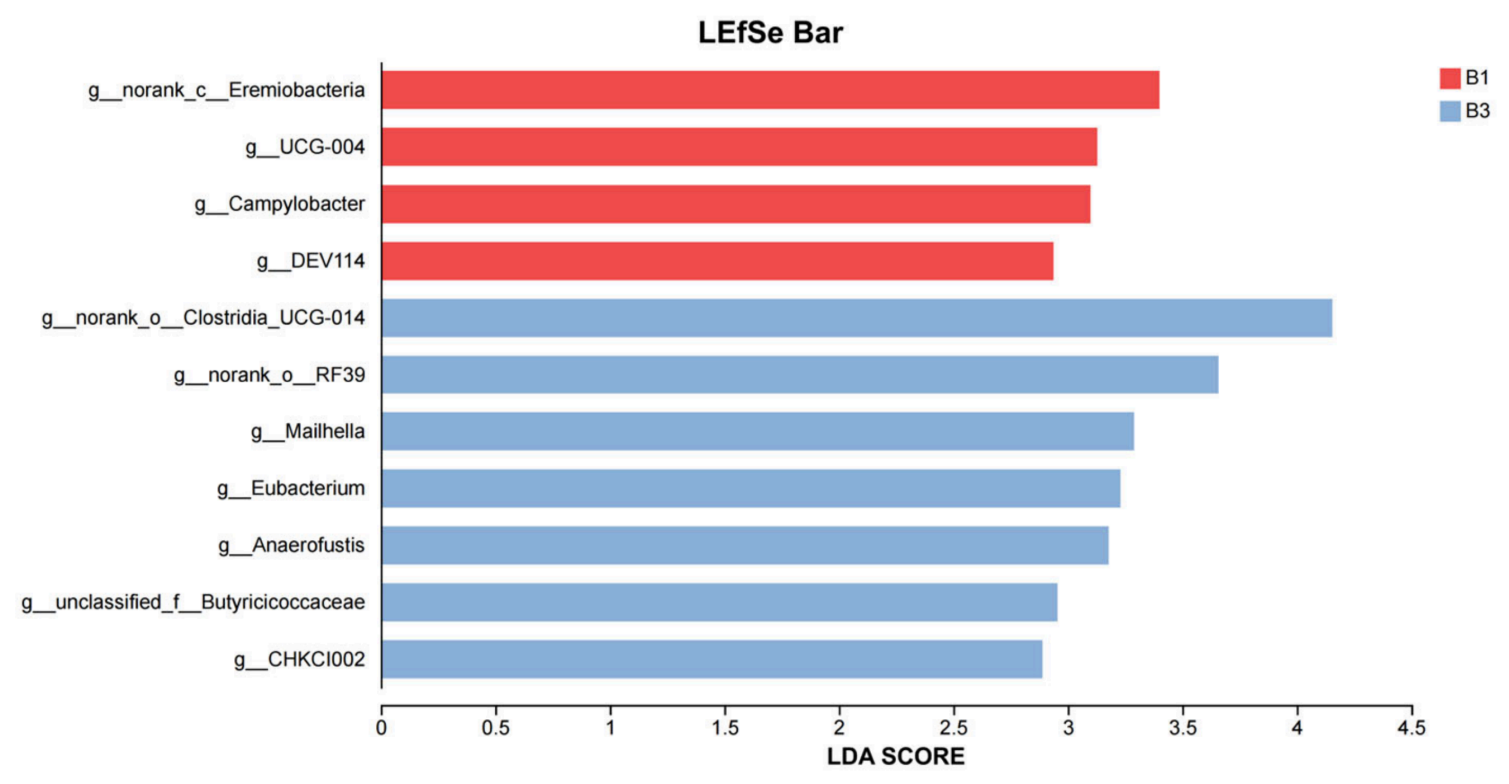
Taxa that were significantly differentially represented between groups were examined by linear discriminant analysis coupled with effect size (LEfSe) using the default parameters (LDA score = 2).

**Figure 7:**
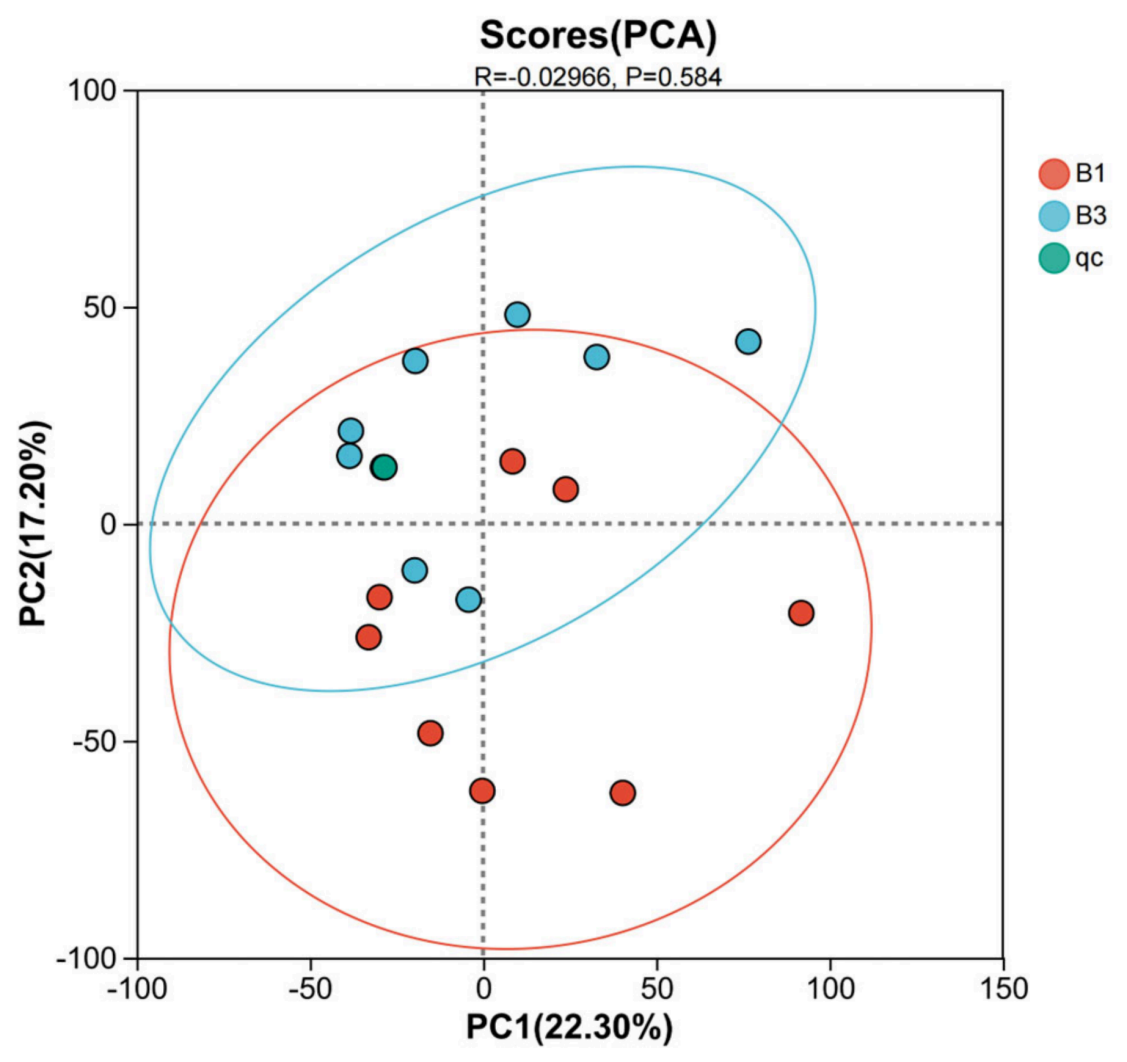
Principal component analysis (PCA) score plot of cecal metabolites in post-peak laying phase.

**Figure 8:**
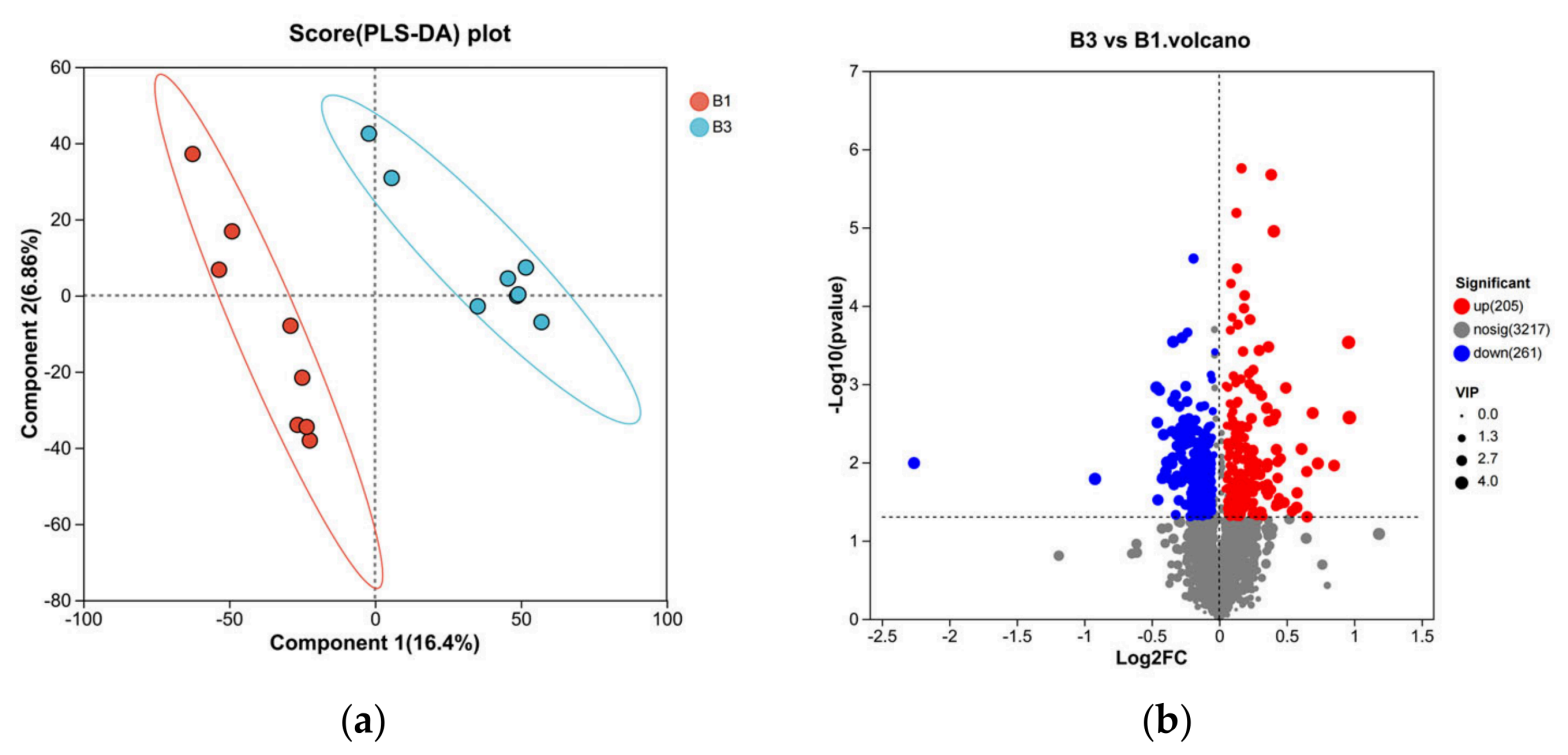
(**a**) Effects of *B. licheniformis* on the metabolome of cecal contents. Partial least squares-discriminant analysis (PLS-DA) score plot. (**b**) Volcano plot of differentially abundant metabolites (DAMs) and Kyoto Encyclopedia of Genes and Genomes (KEGG) pathway enrichment analysis of DAMs in the cecal contents of laying hens fed diet B3 compared with group B1. Red cells indicate a positive correlation (upregulation) and blue cells indicate a negative correlation (downregulation).

**Figure 9:**
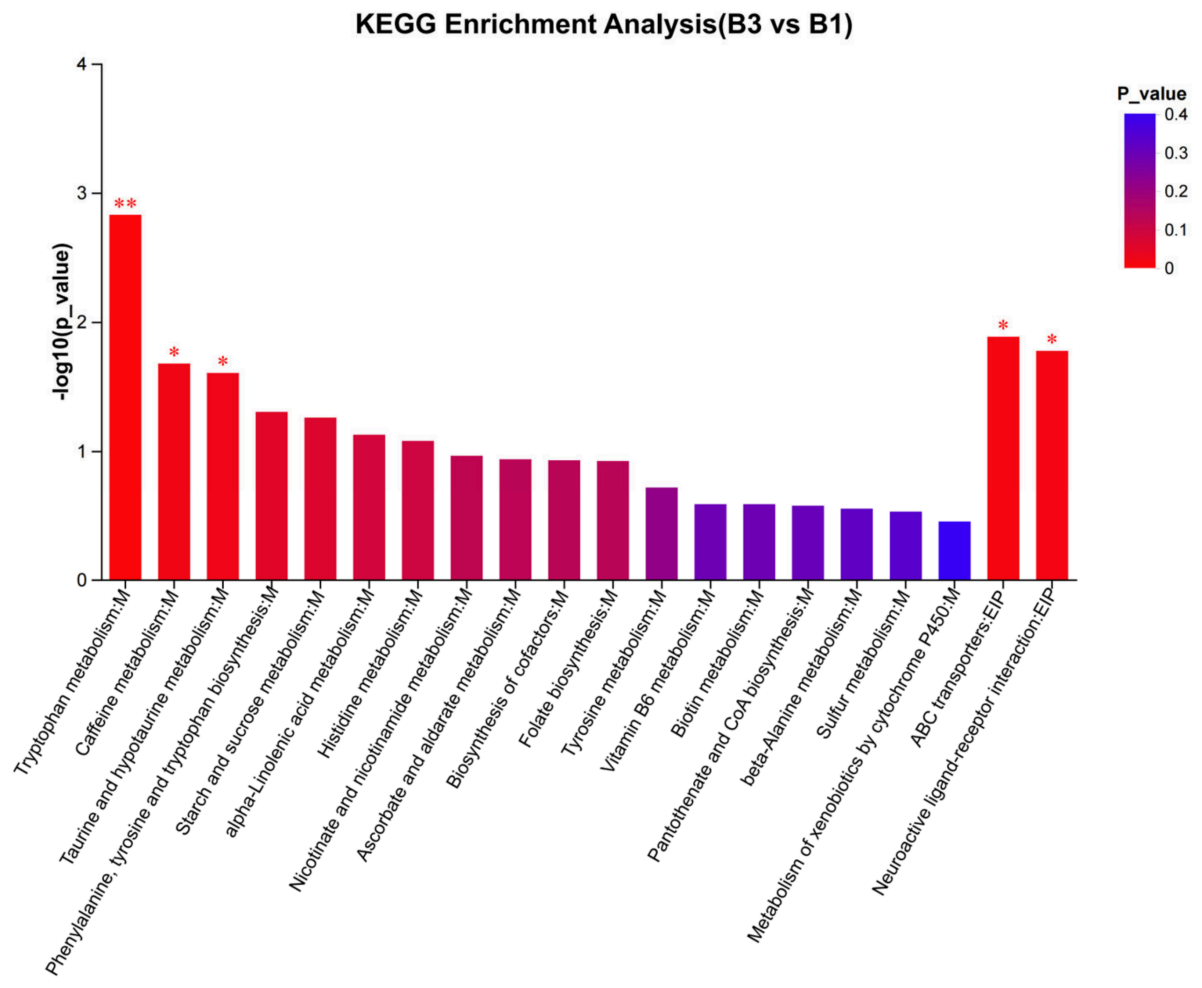
KEGG pathway enrichment analysis of differentially abundant metabolites (DAMs). * *p* < 0.05, ** *p* < 0.01.

**Figure 10:**
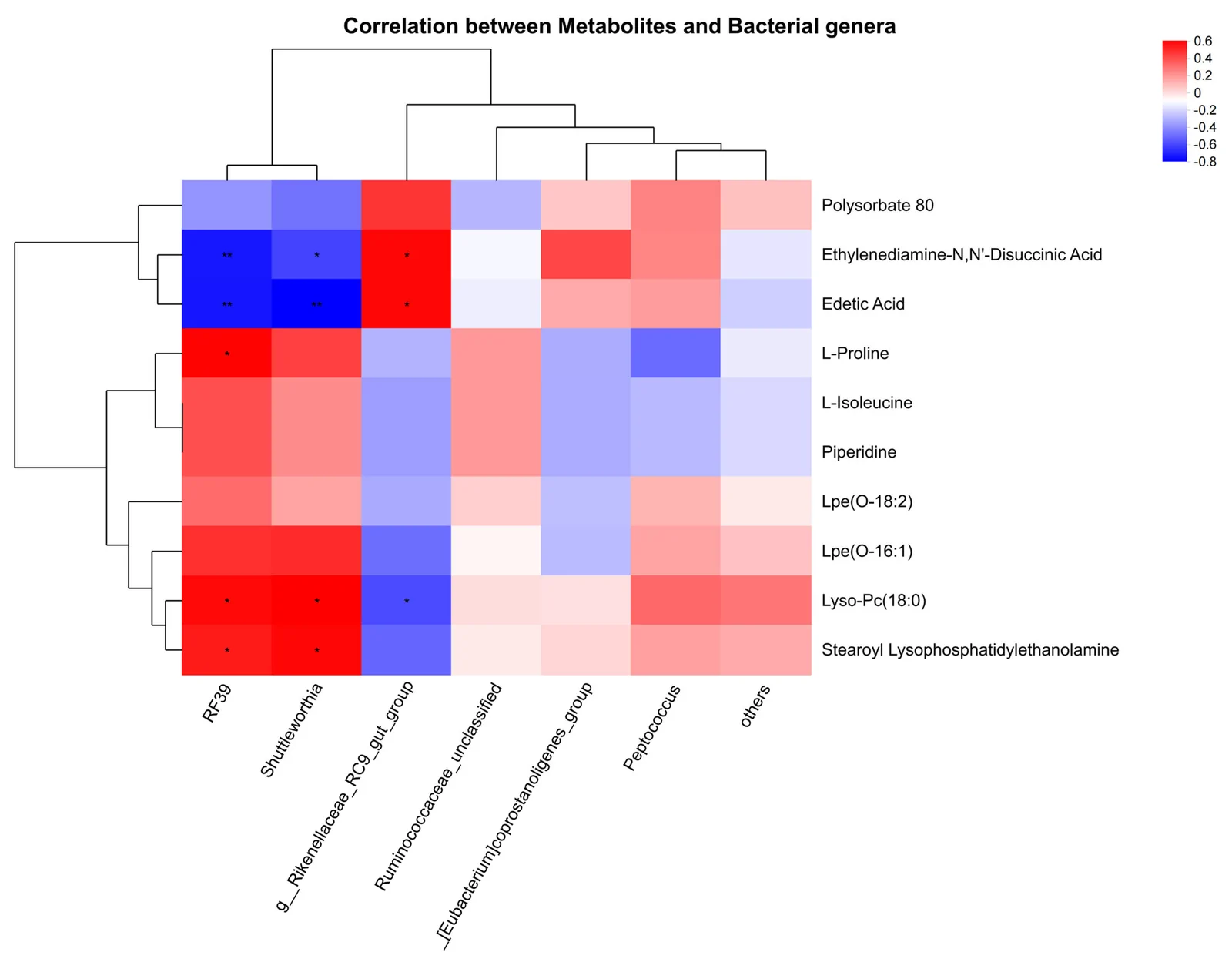
Spearman correlation among cecal top 10 differential metabolites and differential microbiota at genus levels. * *p* < 0.05, ** *p* < 0.01.

**Table 1:** Composition and nutrient levels of basal diet.

Item	Composition (%)
Corn (dehulled)	62
Soybean meal	25
Limestone	9
Compound premix ^1^	4
Total	100
Nutrient levels ^2^	Level
Metabolizable energy (MJ/kg)	11.29
Crude protein (%)	16.20%
Calcium (%)	3.80%
Phosphorus, total (%)	0.40%
Lysine (%)	0.88%
Methionine (%)	0.33%

^1^ The premix provided per kilogram of complete feed contains the following nutrients: Vitamin A: 5360–8800 IU; Vitamin D_3_: 2600–4400 IU; Vitamin E: 72–116 mg; Vitamin K_3_: 1.34–3.00 mg; Vitamin B_1_: 1.28–2.30 mg; Vitamin B_2_: 4.11–7.14 mg; Vitamin B_6_: 2.92–3.12 mg; Vitamin B_12_: 0.012–0.18 mg; biotin: 0.012–0.18 mg; Niacin (nicotinamide): 114.7–230.9 mg; Pantothenic acid: 18.12–29.25 mg; Iron: 6.16 mg; Copper: 2.32 mg; Manganese: 3.12 mg; Zinc: 3.12 mg; Iodine: 0.04 mg; Selenium: 0.016 mg. ^2^ All nutrient levels are calculated values.

**Table 2:** Effects of dietary supplementation with *B. licheniformis* on laying performance of hens.

GraphG1.	B1	B2	B3	B4	*p*-Value
Laying rate (%)	78.96 ± 0.47 ^B^	80.25 ± 0.54 ^AB^	80.40 ± 0.64 ^A^	78.14 ± 0.48 ^B^	0.009
Average egg weight (g)	49.93 ± 0.16 ^B^	50.49 ± 0.15 ^A^	50.02 ± 0.14 ^AB^	50.52 ± 0.13 ^A^	0.006
Feed-to-egg ratio	2.53 ± 0.02 ^A^	2.45 ± 0.02 ^B^	2.47 ± 0.02 ^AB^	2.49 ± 0.02 ^AB^	0.005

Note: data are presented as mean ± standard error of the mean (SEM) (*n* = 8). Within the same item, means with different uppercase letters (A, B) differ significantly at *p* < 0.01.

**Table 3:** Effects of dietary *B. licheniformis* supplementation on serum antioxidant, immune, and inflammatory parameters in laying hens.

Items	B1	B2	B3	B4	*p*-Value
MDA (nmol/mL)	2.15 ± 0.20 ^A^	1.90 ± 0.14 ^A^	1.38 ± 0.08 ^B^	1.93 ± 0.11 ^A^	0.002
T-AOC (U/mL)	30.26 ± 4.66 ^b^	31.09 ± 5.94 ^b^	43.83 ± 4.90 ^a^	32.71 ± 8.62 ^b^	0.416
GSH-Px (U/mL)	641.41 ± 90.15 ^B^	790.87 ± 4.18 ^AB^	854.65 ± 41.37 ^A^	564.41 ± 58.49 ^B^	0.002
SOD (U/mL)	113.58 ± 16.77 ^b^	141.21 ± 11.49 ^b^	177.22 ± 7.35 ^ab^	200.41 ± 26.90 ^a^	0.011
IgG (μg/mL)	237.54 ± 18.28 ^c^	312.52 ± 13.71 ^a^	304.60 ± 21.24 ^b^	281.64 ± 16.51 ^b^	0.040
IgA (μg/mL)	80.13 ± 12.90	124.15 ± 9.84	129.79 ± 13.88	106.41 ± 13.33	0.060
IgM (μg/mL)	71.35 ± 8.02 ^c^	101.91 ± 5.79 ^a^	93.87 ± 6.16 ^ab^	75.30 ± 7.68 ^bc^	0.012
TNF-α (ng/L)	44.75 ± 5.31 ^A^	38.56 ± 3.52 ^A^	24.98 ± 2.09 ^B^	40.14 ± 2.77 ^A^	0.002
IL-1β (ng/L)	22.44 ± 1.34 ^ab^	18.74 ± 2.55 ^b^	18.68 ± 3.00 ^b^	28.12 ± 2.44 ^a^	0.037
IL-6 (pg/mL)	285.13 ± 14.83 ^b^	267.41 ± 14.84 ^b^	278.60 ± 19.95 ^b^	339.74 ± 17.89 ^a^	0.026
IL-10 (ng/mL)	13.43 ± 1.07	16.03 ± 1.04	15.42 ± 0.71	12.38 ± 1.36	0.076

Note: values represent mean ± SEM (*n* = 8). Within the same item, means with different lowercase letters (a, b, c) differ significantly at *p* < 0.05, while those with different uppercase letters (A, B) differ significantly at *p* < 0.01.

**Table 4:** Effects of dietary *B. licheniformis* supplementation on hepatic antioxidant biochemical parameters in laying hens.

Items	B1	B2	B3	B4	*p*-Value
MDA (nmol/g)	2.56 ± 0.12 ^A^	1.89 ± 0.10 ^AB^	1.76 ± 0.17 ^B^	2.27 ± 0.16 ^AB^	0.009
T-AOC (U/g)	13.19 ± 0.94	12.63 ± 2.27	16.70 ± 0.87	15.05 ± 1.01	0.155
SOD (U/g)	130.16 ± 8.25 ^B^	175.76 ± 5.53 ^A^	165.68 ± 7.56 ^A^	149.32 ± 5.94 ^AB^	0.001
GSH-Px (U/g)	222.27 ± 15.92 ^B^	274.04 ± 36.71 ^AB^	334.23 ± 17.54 ^A^	275.36 ± 14.62 ^AB^	0.005

Note: values represent mean ± SEM (*n* = 8). Within the same item, means with different uppercase letters (A, B) differ significantly at *p* < 0.01.

**Table 5:** Effects of dietary *B. licheniformis* HJ0135 supplementation on intestinal structure in 43-week-old laying hens.

Item	Ileum			Jejunal		
	VH	CD	VH/CD	VH	CD	VH/CD
B1	806.83 ± 7.80 ^b^	178.06 ± 3.82	4.62 ± 0.06 ^c^	916.28 ± 15.54 ^b^	177.40 ± 2.12 ^a^	4.646 ± 0.06 ^d^
B2	839.38 ± 10.59 ^b^	173.93 ± 3.70	4.92 ± 0.06 ^b^	1025.81 ± 16.31 ^a^	161.64 ± 2.61 ^b^	6.428 ± 0.12 ^b^
B3	845.14 ± 7.69 ^a^	165.24 ± 2.10	5.15 ± 0.04 ^a^	1040.09 ± 19.99 ^a^	154.05 ± 2.99 ^b^	7.645 ± 0.15 ^a^
B4	818.60 ± 11.19 ^b^	173.24 ± 4.04	4.83 ± 0.06 ^b^	1009.67 ± 22.69 ^a^	172.43 ± 3.44 ^a^	5.795 ± 0.06 ^c^

Note: values represent mean ± SEM (*n* = 8). Different lowercase letters (a, b, c, d) within the same item represent significantly different means (*p* < 0.05).

**Table 6:** Differentially expressed metabolites in cecal contents of laying hens supplemented with *B. licheniformis* HJ0135 compared to the control group.

Ion Mode	All Peaks	Identified Metabolites	Unidentified	Metabolites in Library	Metabolites in KEGG
Positive (pos)	13,858	1866	11,992	1132	514
Negative (neg)	12,599	1817	10,782	1408	585
Total (mix)	26,457	3683	22,774	2540	1099

Note: values represent the total counts of peaks and metabolites detected. Identified metabolites were obtained by matching against the in-house spectral library and the KEGG database. pos, positive ionization mode; neg, negative ionization mode; mix, combined positive and negative modes.

**Table 7:** Differentially expressed cecal metabolites in laying hens supplemented with *B. licheniformis* HJ0135 at 5 × 10^9^ CFU/kg compared with the control group.

Metabolite	*p*-Value	VIP	Log_2_FC	Regulate	HMDB Class	HMDB Subclass
Pc (O-16:0/18:2)	0.003	2.15	0.176	up	-	-
Lpe (18:1)	0.023	1.34	0.064	up	Glycerophospholipids	Glycerophosphoethanolamines
L-Proline	0.002	1.31	0.047	up	Carboxylic acids and derivatives	Amino acids, peptides, and analogs
8,11-Eicosadiynoic Acid	0.039	1.24	0.078	up	Carboxylic acids and derivatives	Amino acids, peptides, and analogs

Note: differentially expressed metabolites were identified using the criteria VIP > 1 and *p* < 0.05. Log_2_FC represents the log_2_ fold change (supplementation/control). HMDB class and subclass were assigned based on the Human Metabolome Database; “-” denotes no available classification. *p* < 0.05, *p* < 0.01 compared with the control group.

## Data Availability

The DNA sequences in this article were deposited in the National Center for Biotechnology Information (NCBI) Sequence Read Archive (SRA) repository under accession number PRJNA1444896.
